# Is lobe specific lymph node dissection adequate for cN0–1 non-small cell lung cancer?

**DOI:** 10.1186/s13019-020-1087-4

**Published:** 2020-02-27

**Authors:** Likui Fang, Jinming Xu, Bo Ye, Guocan Yu, Gang Chen, Jun Yang

**Affiliations:** 1 Department of Thoracic Surgery, Hangzhou Red Cross Hospital, Hangzhou, 310003 China; 2 Department of Thoracic Surgery, the First Affiliated Hospital, Zhejiang University School of Medicine, Hangzhou, 310003 China

**Keywords:** Non-small cell lung cancer, Lymph node metastasis, Lymph node dissection

## Abstract

**Purpose:**

This study aims to explore whether lobe specific lymph node dissection (LND) is adequate for cN0–1 non-small cell lung cancer (NSCLC) or not.

**Methods:**

Among 5613 cN0–1 NSCLC patients, 394 cases (7.0%) with pN2 were enrolled and the distribution of mediastinal lymph node metastasis was analyzed. The included patients were divided into the non-lobe specific lymph node metastasis (NLSLNM) group and the lobe specific lymph node metastasis (LSLNM) group. The clinicopathological characteristics were compared between two groups and multivariable analysis was performed to find independent factors predicting NLSLNM.

**Results:**

The incidence of pN2 cases deserved serious attention. The proportion of upper zone lymph node metastases was not rare in right (55.0%) and left (35.7%) lower lobe tumors. The proportion of subcarinal zone lymph node involvement was also high in right (21.8%) and left (25.8%) upper lobe tumors. Multivariable analysis showed that elevated carcinoembryonic antigen (CEA) level (*P* = 0.034), right lower lobe (RLL) tumors (*P* = 0.022) and station 11 involvement (*P* = 0.030) were independent risk factors for NLSLNM.

**Conclusion:**

Systematic LND seems to be superior to lobe specific LND in the assessment of lymph node status and high CEA level, RLL tumors and station 11 involvement are predictors for NLSLNM.

## Introduction

Non-small cell lung cancer (NSCLC) has been one of the most common malignant tumors and the leading cause of cancer-related deaths in the world [1, 2]. Although the treatment methods and prognosis of NSCLC have been improving [3, 4], there are still many debatable problems. In general, surgery is recommended for the patients with resectable NSCLC, and lobectomy combined with systematic lymph node dissection (LND) is the standard surgical procedure [5]. However, this surgical treatment has been questioned with the increasing preference of minimal invasive surgery. The optimal extent of pulmonary resection has been explored by surgeons constantly, as well as the extent of lymphadenectomy. Sublobar resection including segmentectomy and wedge resection has been proven to be appropriate in patients with early stage NSCLC [6, 7]. In patients with T1-2N0–1M0, mediastinal lymph node sampling has shorter operative time, less chest tube drainage and similar survival outcome compared with complete mediastinal LND [8, 9].

In recent years, selective LND has also raised thoracic surgeons’ interest due to the concept of lobe specific lymphatic metastasis. The pattern of lymph node metastasis is thought to be influenced by the location of primary tumors, with tumors in the upper lobe showing a higher incidence of the superior mediastinal lymph nodes involvement than lower lobe tumors which tend to metastasize to the inferior and subcarinal nodes [10–12]. However, there are still some studies indicating that complete LND is overwhelmingly superior to lobe specific LND from the oncological point of view [13–15]. This study aims to explore whether lobe specific LND is adequate for cN0–1 non-small cell lung cancer (NSCLC) or not.

## Materials and methods

### Patients selection

The study protocol was approved by the Institutional Review Board of Hangzhou Red Cross Hospital and the First Affiliated Hospital of Zhejiang University, School of Medicine. From January 2012 to May 2018, a total of 5613 patients with cN0–1 NSCLC had undergone surgery in our institutions. The data of patients were reviewed retrospectively from hospital electronic medical records systems, including demographic data, preoperative investigations and pathological characteristics.

The patients included in the analysis fitted with the following criteria: (1) the disease was diagnosed as cN0–1 preoperatively but was confirmed as pN2 postoperatively; (2) the patient did not have distant metastasis before treatment; (3) the histology was classified as NSCLC; (4) mediastinal LND was performed together with pulmonary resection. And we excluded patients who had multiple tumors in different lobes, those who received induction therapy preoperatively, including chemotherapy or radiotherapy, and those who underwent only biopsy and selective LND. We also excluded patients with tumors invading multiple lobes. Ultimately, 394 patients were included in this study. All enrolled patients were restaged according to the 8th edition of the American Joint Committee on Cancer (AJCC) lung cancer staging classification [16].

### Staging policy and lymph node assessment

Preoperative stage evaluations included physical examination, chest CT, abdominal ultrasound, magnetic resonance imaging (MRI) of the brain and fiberoptic bronchoscopy. Bone scintigraphy was only performed in the patients with bone pain and positron-emission tomography (PET) scan was also not routinely performed in early stage NSCLC. Clinical lymph node (LN) status was assessed by CT scan, PET scan and endobronchial ultrasound-guided transbronchial needle aspiration (EBUS-TBNA). The lymph node was considered to be positive when its shortest axis was longer than 1 cm on CT scan. PET scan and EBUS-TBNA were not routinely performed unless the patients was highly suspected as N2 disease on CT scan.

The site of mediastinal lymph node was described according to the International Association for the Study of Lung Cancer (IASLC) lymph node map [17], with the upper zone including stations 2, 3 and 4, the aortic-pulmonary zone including stations 5 and 6, the subcarinal zone including station 7 and the lower zone including stations 8 and 9. Lobe specific lymph node stations (LSLNS) depended on the location of primary tumors (stations 2R, 3 and 4R for right upper lobe tumors, stations 4 L, 5 and 6 for left upper lobe tumors, stations 7, 8 and 9 for lower lobe tumors) [18]. Tumors metastasizing to LSLNS was defined as lobe specific lymph node metastasis (LSLNM), while tumors metastasizing beyond LSLNS was defined as non-lobe specific lymph node metastasis (NLSLNM). Skip N2 metastasis was defined as mediastinal lymph node metastasis without N1 metastasis [19].

### Surgical procedures

All patients underwent anatomic pulmonary resection combined with mediastinal LND. Mediastinal LND for right side tumors included at least stations 2R, 4R, 7, 8 and 9, while for left side tumors, stations 5, 6, 7, 8 and 9 were required at least. Due to the anatomic limitations, dissection of station 3 and 4 L was not routinely performed unless the lymph nodes metastases were highly suspicious preoperatively or intraoperatively.

### Statistical analysis

The measurement data and numeration data were statistically analyzed with t test and χ^2^ test respectively. If there were clinicopathological characteristics showing significant differences between the NLSLNM group and the LSLNM group, multivariate analysis was performed for those characteristics by the binary logistic regression to identify the factors predicting NLSLNM. Because the lymphatic metastasis patterns of right middle lobe tumors were unclear, the data of those tumors were excluded from the comparison between NLSLNM group and the LSLNM group. All the above analysis was conducted by SPSS software (version 24.0, IBM SPSS Inc. United States). Statistical significance was set at *P* value < 0.05 (All *P* values presented were 2-sided).

## Results

### Distribution of mediastinal lymph node metastasis

Among 5613 cN0–1 NSCLC patients, 394 cases (7.0%) with pN2 were enrolled, and 213 had right NSCLC while the other 181 had left NSCLC. The distributions of mediastinal lymph node metastasis in right and left sides were listed in Table 1 and Table 2, respectively.

**Table 1:** Mediastinal lymph node metastasis in right-sided NSCLC

Variables	RUL (*n* = 78)	RML (*n* = 35)	RLL (*n* = 100)	*P* value
Upper zone	74/78 (94.9%)	20/35 (57.1%)	55/100 (55.0%)	< 0.001
2R	34/78 (43.6%)	13/35 (37.1%)	14/100 (14.0%)	< 0.001
3	12/29 (41.4%)	5/14 (35.7%)	15/43 (34.9%)	0.848
4R	63/78 (80.8%)	18/35 (51.4%)	43/100 (43.0%)	< 0.001
Subcarinal zone				
7	17/78 (21.8%)	29/35 (82.9%)	86/100 (86.0%)	< 0.001
Lower zone	2/78 (2.6%)	1/35 (2.9%)	13/100 (13.0%)	0.017
8	1/78 (1.3%)	0/35 (0)	9/100 (9.0%)	0.019
9	2/78 (2.6%)	1/35 (2.9%)	5/100 (5.0%)	0.666
Negative LSLNS	4/78 (5.1%)	/	11/100 (11.0%)	0.162

*NSCLC* non-small cell lung cancer; *RUL* right upper lobe; *RML* right middle lobe; *RLL* right lower lobe; *LSLNS* lobe specific lymph node stations

Values are the number of involved patients divided by the number of resected patients (%)

**Table 2:** Mediastinal lymph node metastasis in left-sided NSCLC

Variables	LUL (*n* = 97)	LLL (*n* = 84)	*P* value
Upper zone	84/97 (86.6%)	30/84 (35.7%)	< 0.001
4 L	14/29 (48.3%)	7/22 (31.8%)	0.237
5	71/97 (73.2%)	24/84 (28.6%)	< 0.001
6	27/97 (27.8%)	10/84 (11.9%)	0.008
Subcarinal zone			
7	25/97 (25.8%)	57/84 (67.9%)	< 0.001
Lower zone	4/97 (4.1%)	32/84 (38.1%)	< 0.001
8	1/97 (1.0%)	12/84 (14.3%)	0.001
9	4/97 (4.1%)	27/84 (32.1%)	< 0.001
Negative LSLNS	12/97 (12.4%)	12/84 (14.3%)	0.705

*NSCLC* non-small cell lung cancer; *LUL* left upper lobe; *LLL* left lower lobe; *LSLNS* lobe specific lymph node stations

Values are the number of involved patients divided by the number of resected patients (%)

Right upper lobe (RUL): lymph nodes in upper zone were involved much more often in the RUL compared with the other two lobes (94.9% vs. 57.1% vs. 55.0%, *P* < 0.001). Station 4R (80.8%) had the highest proportion to be involved, followed by station 2R (43.6%) and station 3 (41.4%). Station 7 involvement (21.8%) was also occurred with a relatively high proportion. Lymph nodes in station 8 (1.3%) and station 9 (2.6%) were less likely to be involved than other stations.

Right middle lobe (RML): the highest proportion of metastasis was observed in subcarinal zone, station 7 (82.9%). The involvement of upper zone was common as well, with 51.4% in station 4R, 35.7% in station 3 and 37.1% in station 2R. Only 1 patient (2.9%) had positive lymph nodes in the lower zone.

Right lower lobe (RLL): station 7 (86.0%) was also the most common site to be involved in the RLL, follow by station 4R (43.0%). The involvement of station 2R (14.0%) and station 3 (34.9%) was not rare. The metastasis of RLL to the lower zone was more often than the RUL and RML (13.0% vs. 2.6% vs. 2.9%, *P* = 0.017).

It was notable that 4 patients (5.1%) with RUL tumors had negative LSLNS and 11 patients (11.0%) with RLL tumors had negative LSLNS.

Left upper lobe (LUL): station 5 (73.2%) was the most common site to be involved, followed by station 4 L (48.3%) and 6 (27.8%). The involvement of station 7 (25.8%) was also not rare. As was expected, metastases in the lower zone lymph nodes occupied small percentage, with only 4.1%.

Left lower lobe (LLL): the highest proportion of metastasis was observed in station 7 (67.9%), far more common than the LUL (*P* < 0.001). It should be noted that the proportion of the upper zone lymph nodes involvement (35.7%) was not far less than that of the lower zone involvement (38.1%).

It should be emphasized that there were 12.4 and 14.3% pN2 patients having negative LSLNS in the LUL and LLL, respectively.

### Characteristics of patients with NLSLNM and LSLNM

All enrolled patients except those with RML tumors were divided into the NLSLNM group and LSLNM group on the basis of the lymphatic metastasis pattern, with 129 in NLSLNM group and 230 in LSLNM group. Clinical and pathological characteristics of two groups were shown in Table 3 and Table 4.

**Table 3:** Clinical characteristics of patients with NLSLNM and LSLNM

Variables	NLSLNM (*n* = 129)	LSLNM (*n* = 230)	*P* value
Sex			0.149
Male	61 (47.3%)	127 (55.2%)	
Female	68 (52.7%)	103 (44.8%)	
Age, years			0.176
Median (range)	61 (26–83)	60.5 (27–80)	
Smoking history	42 (32.6%)	115 (50.0%)	0.001
Hypertension	21 (16.3%)	66 (28.7%)	0.008
Diabetes	8 (6.2%)	14 (6.1%)	0.965
Other pulmonary diseases	5 (3.9%)	5 (2.2%)	0.544
Other malignant tumors within 5 years	0 (0)	4 (1.7%)	0.326
CEA level			0.004
>5 ng/ml	65 (50.4%)	80 (34.8%)	
≤5 ng/ml	64 (49.6%)	150 (65.2%)	
Tumor location			< 0.001
RUL	19 (14.7%)	59 (25.7%)	
RLL	54 (41.9%)	46 (20.0%)	
LUL	26 (20.2%)	71 (30.9%)	
LLL	30 (23.3%)	54 (23.5%)	
CT characteristics			0.404
GGO	1 (0.8%)	1 (0.4%)	
Partially solid	4 (3.1%)	14 (6.1%)	
Solid	124 (96.1%)	215 (93.5%)	
Clinical T stage			0.493
T1a	2 (1.6%)	4 (1.7%)	
T1b	19 (14.7%)	49 (21.3%)	
T1c	47 (36.4%)	70 (30.4%)	
T2a	27 (20.9%)	56 (24.3%)	
T2b	20 (15.5%)	27 (11.7%)	
T3	13 (10.1%)	19 (8.3%)	
T4	1 (0.8%)	5 (2.2%)	
Clinical N stage			0.647
N0	118 (91.5%)	207 (90.0%)	
N1	11 (8.5%)	23 (10.0%)	
Surgical approach			0.052
Thoracotomy	71 (55.0%)	102 (44.3%)	
VATS	58 (45.0%)	128 (55.7%)	
Surgical resection			0.345
Segmentectomy	2 (1.6%)	6 (2.6%)	
Lobectomy	120 (93.0%)	207 (90.0%)	
Sleeve lobectomy	2 (1.6%)	11 (4.8%)	
Pneumonectomy	5 (3.9%)	6 (2.6%)	

*NLSLNM* non-lobe specific lymph node metastasis; *LSLNM* lobe specific lymph node metastasis; *CEA* carcinoembryonic antigen; *RUL* right upper lobe; *RLL* right lower lobe; *LUL* left upper lobe; *LLL* left lower lobe; *GGO* ground-glass opacity; *VATS* video assisted thoracic surgery

Values are N (percentage) unless otherwise specified

**Table 4:** Pathological characteristics of patients with NLSLNM and LSLNM

Variables	NLSLNM (*n* = 129)	LSLNM (*n* = 230)	*P* value
Anatomical type			0.168
Central	31 (24.0%)	71 (30.9%)	
Peripheral	98 (76.0%)	159 (69.1%)	
Histology			0.022
Adenocarcinoma	103 (79.8%)	161 (70.0%)	
SCC	14 (10.9%)	52 (22.6%)	
Others	12 (9.3%)	17 (7.4%)	
Cell differentiation			0.480
Well-moderate	3 (2.3%)	2 (0.9%)	
Moderate	25 (19.4%)	56 (24.3%)	
Moderate-poor	62 (48.1%)	111 (48.3%)	
Poor	33 (25.6%)	52 (22.6%)	
NA^a^	6 (4.7%)	9 (3.9%)	
Pathological T stage			0.886
T1a	1 (0.8%)	2 (0.9%)	
T1b	17 (13.2%)	31 (13.5%)	
T1c	24 (18.6%)	33 (14.5%)	
T2a	54 (41.9%)	112 (48.7%)	
T2b	17 (13.2%)	24 (10.4%)	
T3	12 (9.3%)	21 (9.1%)	
T4	4 (3.1%)	7 (3.0%)	
Pathological TNM stage			0.853
IIIA	113 (87.6%)	203 (88.3%)	
IIIB	16 (12.4%)	27 (11.7%)	
Tumor size^b^, cm			0.830
Median (range)	3.0 (0.8–8.0)	3.0 (0.9–10.0)	
Visceral pleural invasion	51 (39.5%)	73 (31.7%)	0.136
Lymphovascular invasion	30 (23.3%)	44 (19.1%)	0.354
Skip N2 metastasis	25 (19.4%)	65 (28.3%)	0.062
N1 involvement			
10	51 (39.5%)	61 (26.5%)	0.011
11	47 (36.4%)	48 (20.9%)	0.001
12	83 (64.3%)	124 (53.9%)	0.055

*NLSLNM* non-lobe specific lymph node metastasis; *LSLNM* lobe specific lymph node metastasis; *SCC* Squamous cell carcinoma; *NA* not available

^a^ Cases that were not available, and were hence excluded when calculating the *p*-value

^b^ Tumor size was defined as the maximum diameter of the pathological specimens

Values are N (percentage) unless otherwise specified

There were no statistical differences in sex and age between two groups. In contrast, more patients in LSLNM group had smoking history (50.0% vs. 32.6%, *P* = 0.001) and hypertension (28.7% vs. 16.3%, *P* = 0.008) than those in NLSLNM group. Abnormally elevated carcinoembryonic antigen (CEA) level (>5 ng/ml) was detected in more patients in NLSLNM group than LSLNM group (50.4% vs. 34.8%, *P* = 0.004). The NLSLNM group significantly tended to have RLL tumors while the LSLNM group was more likely to have upper lobe tumors (*P* < 0.001). The proportion of solid tumors presenting on CT scan was comparable at approximately 95% in both groups. It was worth mentioning that there was one patient in both groups presenting ground-glass opacity (GGO) on CT scan. Clinical T stage and N stage were also similar in two groups.

There were no significant differences in pathological characteristics, except for histology and N1 involvement. Adenocarcinoma was observed in greater percentage of patients in NLSLNM group while squamous cell carcinoma was detected more often in LSLNM group (*P* = 0.022). Station 10 and 11 lymph nodes were less likely to be involved in LSLNM group compared with those in NLSLNM group (26.5% vs. 39.5%, *P* = 0.011 and 20.9% vs. 36.4%, *P* = 0.001, respectively).

### Factors predicting NLSLNM

The univariate analysis showed that smoking history, hypertension, CEA level, tumor location, histology, stations 10 and 11 involvement were statistically significant factors influencing the lymphatic metastasis pattern. Multivariate analysis was further performed for these factors (Table 5). The results indicated that hypertension (*P* = 0.025), CEA level (*P* = 0.034), tumor location (*P* = 0.022) and station 11 involvement (*P* = 0.030) were statistically associated with NLSLNM. High CEA level (OR = 1.684, 95% CI = 1.040–2.725), RLL tumors (OR = 2.111, 95% CI = 1.116–3.992) and station 11 involvement (OR = 1.774, 95% CI = 1.056–2.980) were independent risk factors for NLSLNM, while hypertension (OR = 0.512, 95% CI = 0.286–0.918) was a protective factor for NLSLNM.

**Table 5:** Independent predictive factors of non-lobe specific lymph node metastasis

Variables	*P* value	OR	95% CI
Hypertension	0.025	0.512	0.286–0.918
Smoking history	0.077	0.617	0.361–1.053
CEA level	0.034	1.684	1.040–2.725
Tumor location			
RUL	0.165	0.598	0.289–1.236
RLL	0.022	2.111	1.116–3.992
LUL	0.335	0.717	0.364–1.411
LLL	/	1	/
Histology			
Adenocarcinoma	/	1	/
SCC	0.487	0.759	0.348–1.654
Others	0.733	1.162	0.491–2.749
N1 involvement			
10	0.152	1.446	0.873–2.396
11	0.030	1.774	1.056–2.980

*OR* odds ratio; *CI* confidence interval; *CEA* carcinoembryonic antigen; *RUL* right upper lobe; *RLL* right lower lobe; *LUL* left upper lobe; *LLL* left lower lobe; *SCC* Squamous cell carcinoma

## Discussion

Although an increasing number of lung cancers are discovered in early stage with the development of lung cancer screening, there have been several studies reporting that unsuspected N2 disease was diagnosed in 4.4–9% of clinical stage I–II NSCLC [15, 20]. The lymph node status including hilar and mediastinal lymph nodes has been one of the most important factors to determine the need for adjuvant therapies and predict the prognosis [21]. Systematic LND that dissects the hilar and mediastinal lymph nodes completely could not only guarantee the accuracy of N stage but also guide the postoperative treatment strategy precisely. As a result, systematic LND has been recommended along with lobectomy, regardless of the tumor stage and location. However, on the basis of lobe specific nodal drainage pattern, lobe specific LND has appealed to some surgeons. Aokage, K. et al. [11] suggested that it was safe to omit station 7 dissection in upper lobe NSCLC patients because subcarinal node metastases from upper lobe NSCLC were rare. Adachi, H. et al. [22] indicated that lobe specific LND might be a standard procedure in surgical treatment for cT1-2 N0–1 M0 NSCLC due to the similar 5-year overall survival between the lobe specific LND group and the systematic dissection group. Also, the most recent and largest retrospective registry study showed that lobe specific LND did not have a negative prognostic impact and it had the potential to be an alternative to systematic nodal dissection for the patients with stage I or II NSCLC [23].

However, several publications suggested that tumor locations were not the predictor of lymphatic drainage pathways [13] and there was a considerable number of pN2 patients having mediastinal lymph node metastasis beyond the lobe specific lymph node stations [15], so systematic LND was still recommended to be performed, even in clinical stage I NSCLC. There are two main reasons for the ambiguous role of lobe specific LND. First, the similar impact on long-term survival between lobe specific LND and systematic LND has not been confirmed by a prospective randomized study. Fortunately, a multi-institutional and randomized Phase III trial (JCOG1413) began in January 2017 to confirm the clinical benefit in terms of survival non-inferiority and less invasiveness of lobe specific LND compared with systematic LND in patients with clinical stage I–II NSCLC [18]. Secondly, the factors predicting NLSLNM or LSLNM has remained unclear.

This study focused on the association between the clinicopathological characteristics and mediastinal lymphatic metastasis pattern in cN0–1 NSCLC. We found that abnormally elevated CEA level (>5 ng/ml), RLL tumors and station 11 involvement were independent risk factors for NLSLNM. This study also explored the distributions of mediastinal lymph node metastasis. Our findings showed that mediastinal lymph node metastasis pattern conformed to “lobe specific” rule to some extent. For example, the upper zone lymph nodes were more likely to be involved in the upper lobe tumors, while the subcarinal zone lymph nodes were more likely to be involved in the lower lobe tumors. However, the proportion of upper zone lymph node metastases was not rare in lower lobe tumors, and similarly, the proportion of subcarinal zone lymph node involvement in upper lobe tumors was also high enough to warrant attention. Furthermore, LSLNS were negative in more than 5% of each lobe tumors and the high proportion required attention in the selection of lymphadenectomy extent. Lobe specific LND should be performed prudently and systematic LND might be a better procedure. These findings were in line with the results of a previous study including a total of 4511 cases [24].

Our study had two main limitations. First, this was a retrospective study, so the selective bias was inevitable. The results should be confirmed by prospective randomized studies in the future. Secondly, dissection of station 3 and 4 L was not routinely performed and the samples were not large. The pattern of station 3 and 4 L metastases needed to be further explored in future studies.

## Conclusions

In patients with cN0–1 NSCLC, once mediastinal lymph node metastasis occurs, although different primary tumor locations have a different propensity to be sites of mediastinal lymph node metastasis, each zone and each station have relatively high risk to be involved, so systematic LND should be recommended to guarantee the adequate assessment of mediastinal lymph node status. And abnormally elevated CEA level (>5 ng/ml), RLL tumors and station 11 involvement are independently associated with greater risk of non-lobe specific lymph node metastasis in cN0–1pN2 patients.

## Data Availability

The datasets used and/or analyzed during the current study are available from the corresponding author on reasonable request.

## Abbrevations

NSCLC: non-small cell lung cancer
LND: lymph node dissection
NLSLNM: non-lobe specific lymph node metastasis
LSLNM: Lobe specific lymph node metastasis
CEA: Carcinoembryonic antigen
AJCC: American Joint Committee on Cancer
MRI: Magnetic resonance imaging
PET: Positron-emission tomography
EBUS: TBNA endobronchial ultrasound-guided transbronchial needle aspiration
IASLC: International Association for the Study of Lung Cancer
LSLNS: Lobe specific lymph node stations
RUL: Right upper lobe
RML: Right middle lobe
RLL: Right lower lobe
LUL: Left upper lobe
LLL: Left lower lobe
GGO: Ground-glass opacity
VATS: Video assisted thoracic surgery
SCC: Squamous cell carcinoma
NA: Not available
OR: Odds ratio
CI: Confidence interval
